# A Curated Phenotype-Linked HIV-1 Sequence Dataset for Machine Learning Approaches to Tropism Prediction, Disease Progression and Proviral Reservoir Studies

**DOI:** 10.3390/v18101139

**Published:** 2026-10-14

**Authors:** Kirill Elfimov, Alina Nokhova, Ludmila Gotfrid, Maria Gashnikova, Arina Kiryakina, Aleksey Totmenin, Aleksandr Agaphonov, Natalya Gashnikova

**Affiliations:** 1 State Research Centre of Virology and Biotechnology ‘Vector’, Koltsovo 630559, Russia; 2 Federal Research Center for Fundamental and Translational Medicine, Novosibirsk 630060, Russia; 3 Department of Natural Sciences, Novosibirsk State University, Novosibirsk 630090, Russia

**Keywords:** HIV-1, coreceptor tropism, CCR5, CXCR4, V3 region, *env* gene, phenotypic methods, dataset, genotypic tropism prediction, proviral reservoirs

## Abstract

HIV-1 tropism toward the CCR5 and CXCR4 coreceptors is an important viral characteristic associated with cell entry, the potential use of specific entry inhibitors, studies of disease progression, and analyses of proviral reservoirs. The development of tools for genotypic tropism prediction also requires annotated sequence datasets with reliable phenotypic annotation. In this study, we describe a curated HIV-1 sequence dataset intended to complement, rather than replace, existing reference collections. Publications and GenBank records were searched manually using PubMed, Google Scholar, OpenAlex, cross-references, and records from the GenBank Nucleotide database. Sequences were included if a GenBank accession number could be linked to the V3 region of the HIV-1 *env* gene, or to a longer fragment containing this region, and to phenotypically determined tropism; records already present in LANL were removed. The final dataset comprises 1809 records associated with 32 unique PubMed-indexed publications. Subtype B predominated in the dataset; however, non-B variants accounted for 455 records (25.2%), including several Circulating Recombinant Forms (CRFs) and Unique Recombinant Forms (URFs). R5 variants accounted for 1528 records (84.5%), whereas X4 and R5/X4 variants accounted for 281 records (15.5%). The dataset includes 14 categories of phenotypic assays, geographic metadata, and sequences ranging from *env* gene fragments to near-complete HIV-1 genomes. This resource can be used as a complement to existing databases for the development, testing, and external validation of approaches for HIV-1 tropism prediction.

**Dataset:** Zenodo at https://zenodo.org/records/23163974 (accessed on 1 October 2026), reference number (DOI) 10.5281/zenodo.23163974.

**Dataset License:** CC0 1.0 Universal (CC0 1.0) Public Domain Dedication.

## 1. Summary

HIV-1 virion entry into a target cell is a multistep process involving the sequential interaction of the viral envelope protein Env, specifically gp120, with the CD4 receptor and one of the major coreceptors, CCR5 or CXCR4 [1]. Depending on the coreceptor used, viral variants are classified into several tropism categories: R5 variants use the CCR5 coreceptor, X4 variants use the CXCR4 coreceptor, and R5/X4 variants are dual/mixed-tropic and can use both coreceptors, although they usually show a preference for one of them. The V3 loop of gp120 is considered the main genetic determinant of this phenotype, although other regions of the envelope protein also contribute [2]. During ongoing HIV-1 infection and viral transmission, R5 variants usually predominate, whereas the emergence of X4 variants is more commonly observed at later stages of disease [3,4].

Interest in tropism research increased substantially after the introduction of entry inhibitors, particularly CCR5 antagonists [5]. Maraviroc, the first member of this class approved by the Food and Drug Administration (FDA), is effective only against HIV-1 strains with R5 tropism. Therefore, before its administration, viral coreceptor use must be determined and the presence of X4 or R5/X4 variants must be excluded [6,7]. In addition, CCR5 antagonists may select pre-existing X4-tropic quasispecies. In some cases, the virus may also adapt to use the CCR5 coreceptor when it is bound to an antagonist, for example in the presence of mutations conferring resistance to maraviroc [8,9]. Thus, tropism is not only a fundamental viral characteristic, but also a direct factor determining the applicability of HIV entry inhibitor therapy.

Beyond its therapeutic relevance, tropism is closely associated with the clinical course of HIV infection. The emergence of X4 variants has been associated with a more rapid decline in CD4+ T-cell counts, increased cytopathicity, and the development of AIDS-associated diseases [4,10]. At the same time, the causal relationship remains debated. It is not entirely clear whether a shift in tropism accelerates disease progression or instead represents a consequence of immunological changes at late stages of infection, such as a decline in target cells expressing CCR5. Tropism is also relevant to studies of proviral reservoirs. Data on CCR5 antagonists indicate that, under selective pressure, archived minor X4 variants present before therapy may be reactivated or become detectable.

These issues underscore the importance of available data, particularly annotated nucleotide or amino acid sequence datasets in which molecular data are linked to phenotype and, where possible, to clinical context. Phenotypically characterized nucleotide sequences of the HIV-1 *env* gene are of interest not only for selecting CCR5-targeted therapy, but also for analyzing archived viral diversity, including studies of proviral DNA and proviral reservoirs [8], that is, infected cells carrying HIV proviral DNA [11]. In this context, databases in which genotype is directly linked to phenotypic tropism can serve as a basis both for fundamental research and for the development of computational models for in silico genotypic prediction of HIV tropism from nucleotide or amino acid sequences.

The tropism of HIV isolates can be determined using phenotypic or genotypic methods. Phenotypic assays are considered the most accurate, but they are expensive, slow, and technically complex. By contrast, genotypic methods are more accessible and scalable because they rely on sequence data, most often from the V3 region of the *env* gene [2,12,13]. However, existing algorithms, such as Geno2Pheno [14] and WebPSSM [15], show variable accuracy across different HIV-1 genetic variants [16] including Circulating Recombinant Forms (CRFs) and Unique Recombinant Forms (URFs). In particular, systematic discrepancies between phenotype and genotypic prediction have been reported for locally prevalent subtypes and certain recombinant forms, especially CRF01_AE and CRF02_AG. These discrepancies often involve underestimation of X4 tropism [2]. This highlights the need to expand and update sets of phenotypically confirmed sequences suitable for training and external validation of new predictive tools, including machine learning models.

For many years, the Los Alamos National Laboratory (LANL) HIV Sequence Database has remained the main database of annotated HIV sequences. It is a large, long-standing open resource used for the global aggregation and analysis of HIV sequences and associated annotations [17]. According to published descriptions of the project and its subsequent use, this resource has become a de facto reference infrastructure for many areas of HIV research, including viral evolution, immunology, and vaccine development [18,19].

In the field of tropism, LANL serves as the main public reference set of phenotypically characterized sequences [2,20,21]. Nevertheless, no resource is exhaustive. Phenotypically characterized sequences continue to be published and are sometimes not incorporated from the GenBank database for several reasons, including the absence of a clear tropism annotation in the GenBank record, ambiguous interpretation of the record, or the use of non-standard tropism nomenclature. Based on this, the aim of the present study was to create a non-competing, complementary, non-redundant dataset of phenotypically annotated HIV-1 sequences that are absent from the main public LANL search. Expanding such a resource may be useful for the development of genotypic tropism prediction tools, for studies linking tropism to disease progression, and for research related to the HIV-1 proviral landscape.

## 2. Data Description

### 2.1. General Description of the Dataset

The final dataset, obtained after manual verification and removal of sequences present in the LANL database, included 1809 records with unique GenBank accession numbers. In subsequent analyses, each row of the table was treated as the unit of analysis, because each record was linked to a specific GenBank-annotated sequence and its corresponding metadata. For each record, the main fields required for downstream analysis were retained: HIV-1 genetic variant, GenBank accession number, sequence length, sequence or isolate name, PMID and publication title where available, tropism type, phenotypic assay type, authors of the publication or GenBank record, geographic origin of the sample, and the list of HIV-1 genes sequenced within the record. Thus, the final resource was organized as a sequence-oriented table in which molecular data are linked to phenotypic tropism annotation and to available publication, geographic, and genomic metadata.

Most records were linked to a published source indexed in PubMed: a PMID was available for 1527 records (84.4%), whereas 282 records (15.6%) had no PMID. Overall, the dataset included records associated with 32 unique publications. A more detailed description of the dataset is provided in Table 1.

### 2.2. Genetic Composition of the Records

**Figure 1:**
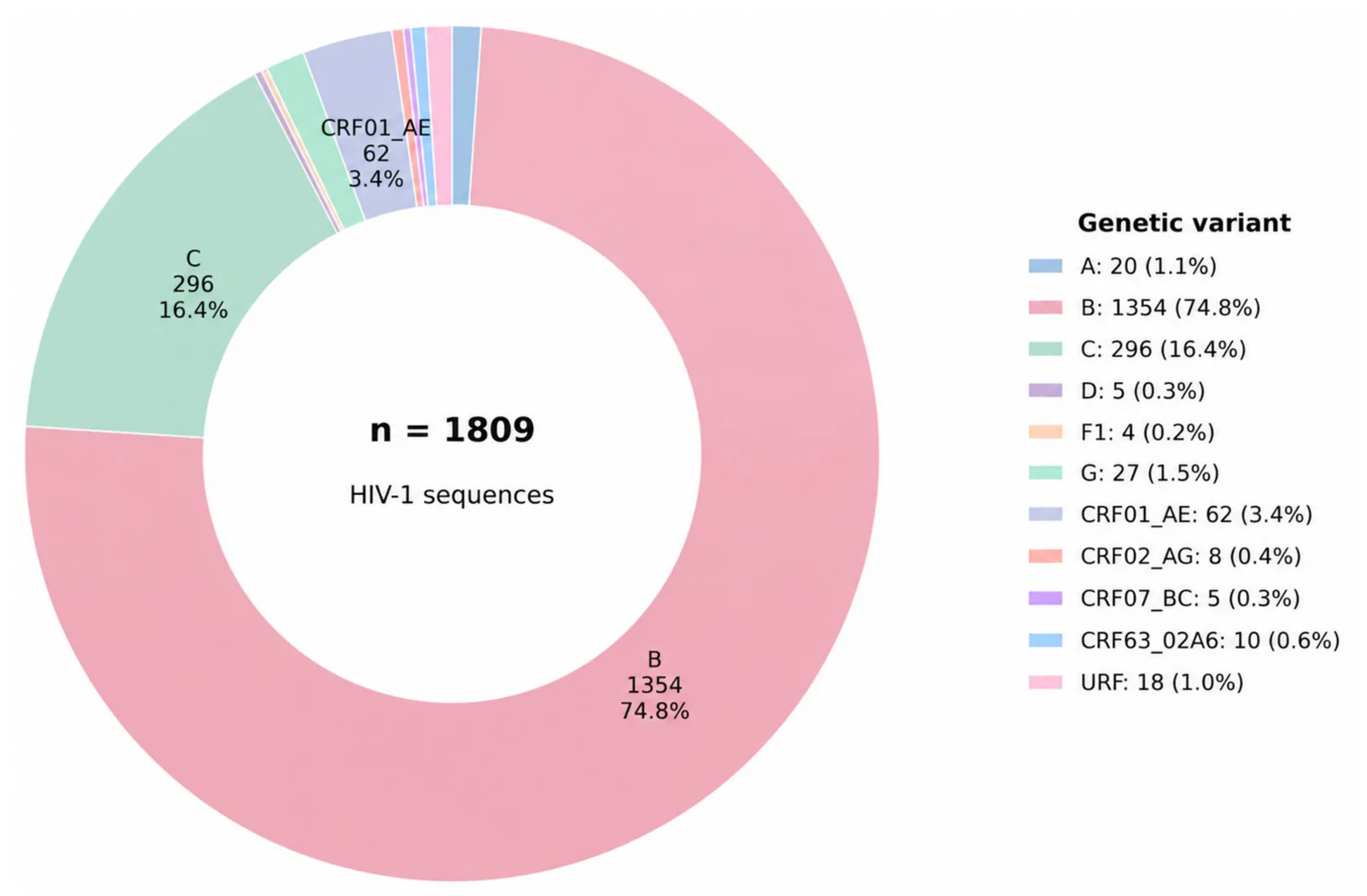
Distribution of HIV-1 genetic variants in the dataset of HIV-1 isolate sequences with tropism determined by phenotypic methods. The donut chart shows the absolute number and proportion of sequences belonging to each subtype, CRF, or URF among the 1809 records in the final dataset. All URF variants were combined into a single “URF” category. Subtype B sequences predominated in the dataset; however, one quarter of the dataset was represented by non-B HIV-1 variants, including subtypes A, C, D, F1, and G, as well as several CRFs and URFs.

In terms of genetic composition, the dataset included both subtype B and several non-B HIV-1 variants, including locally prevalent subtypes (A, F, and G), as well as CRFs and URFs (Figure 1).

The largest group consisted of subtype B sequences, with 1354 records (74.8%). The second largest group was subtype C, with 296 records (16.4%). The dataset also included CRF01_AE, with 62 records (3.4%); subtype G, with 27 records (1.5%); subtype A, with 20 records (1.1%); CRF63_02A6, with 10 records (0.6%); CRF02_AG, with 8 records (0.4%); and rarer subtypes and recombinant forms. Overall, non-B HIV-1 genetic variants accounted for 455 records (25.2%) of the final dataset. CRFs and URFs were represented by 103 records (5.7%) and included CRF01_AE, CRF02_AG, CRF07_BC, CRF63_02A6, as well as several URF variants, including URF_02A3, URF_0209, URF_01B, URF_BF1, URF_A6/B, URF_0109A3, URF_12B, URF_45A1, URF_BG, and URF_63/A6.

### 2.3. Identified Tropism Variants

**Figure 2:**
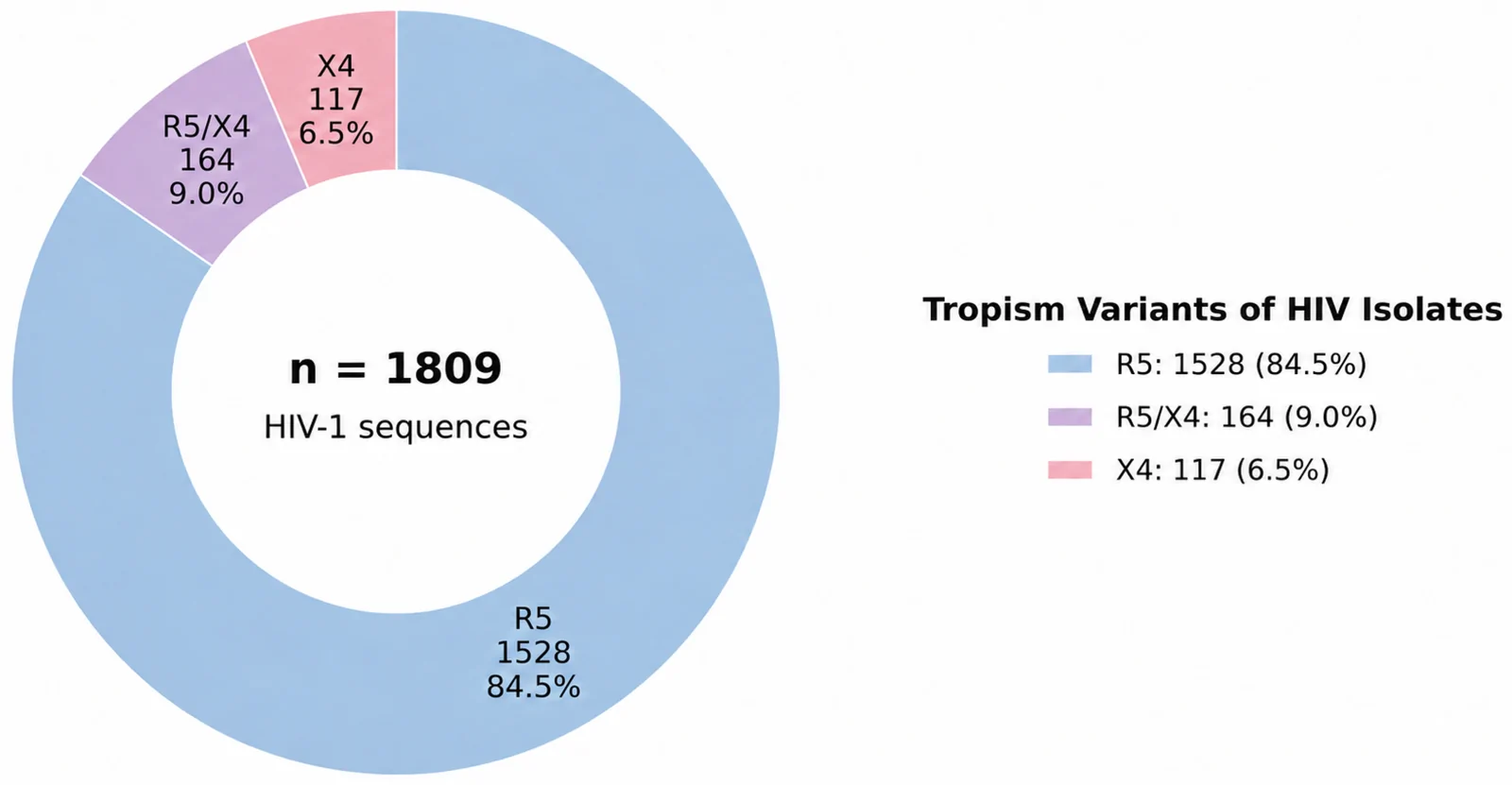
Distribution of HIV-1 sequences by phenotypic tropism in the final dataset. The donut chart shows the absolute number and proportion of records assigned to the R5, R5/X4, and X4 tropism categories. The total number of records in the dataset was *n* = 1809. Sequences with R5 tropism predominated in the dataset (84.5%), whereas variants using the CXCR4 coreceptor, including X4 and R5/X4 variants, represented a smaller proportion of records (15.5%). Percentages were calculated from absolute counts and rounded independently to one decimal place; therefore, summed rounded percentages may differ slightly from percentages calculated for combined categories.

The distribution of phenotypic tropism reflected the predominance of isolates with R5 tropism. This category included 1528 records (84.5%), whereas isolates with R5/X4 tropism accounted for 164 records (9.0%) and X4-tropic isolates accounted for 117 records (6.5%; Figure 2). Thus, variants using the CXCR4 coreceptor, including X4 and R5/X4 variants, comprised 281 records (15.5%) of the final dataset.

**Figure 3:**
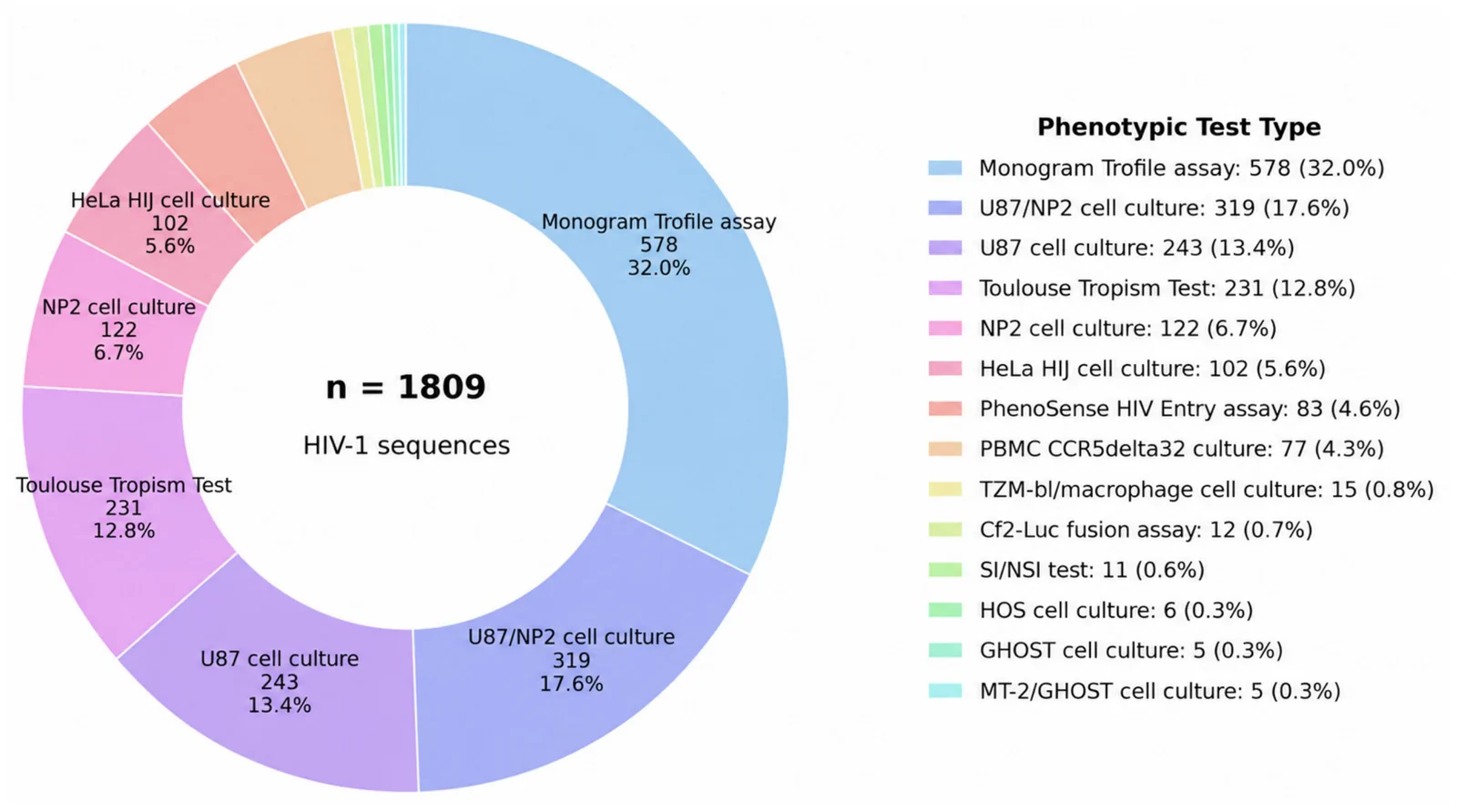
Distribution of dataset records by the type of phenotypic assay used to determine HIV-1 tropism. The donut chart shows the absolute number and proportion of records associated with each type of phenotypic assay. The dataset included both commercial phenotypic assays, including the Monogram Trofile assay and PhenoSense HIV Entry assay, and cell-based systems using U87, NP2, HeLa HIJ, GHOST, MT-2/GHOST, PBMC CCR5Δ32, and other models.

To obtain phenotypic tropism information for the isolates, various experimental approaches described in the cited publications were analyzed. In total, 14 categories of phenotypic assays were identified. The most frequent categories were the Monogram Trofile assay, with 578 records (32.0%); combined use of U87 and NP2 cell cultures, with 319 records (17.6%); U87 cell culture, with 243 records (13.4%); the Toulouse Tropism Test, with 231 records (12.8%); NP2 cell culture, with 122 records (6.7%); HeLa HIJ cell culture, with 102 records (5.6%); the PhenoSense HIV Entry assay, with 83 records (4.6%); and primary peripheral blood mononuclear cell (PBMC) culture with the CCR5Δ32 mutation, with 77 records (4.3%; Figure 3). Together, these eight most common categories covered 1755 records (97.0%) of the final dataset, whereas the remaining methods were represented by fewer records.

### 2.4. Geographic Origin of the Samples

**Figure 4:**
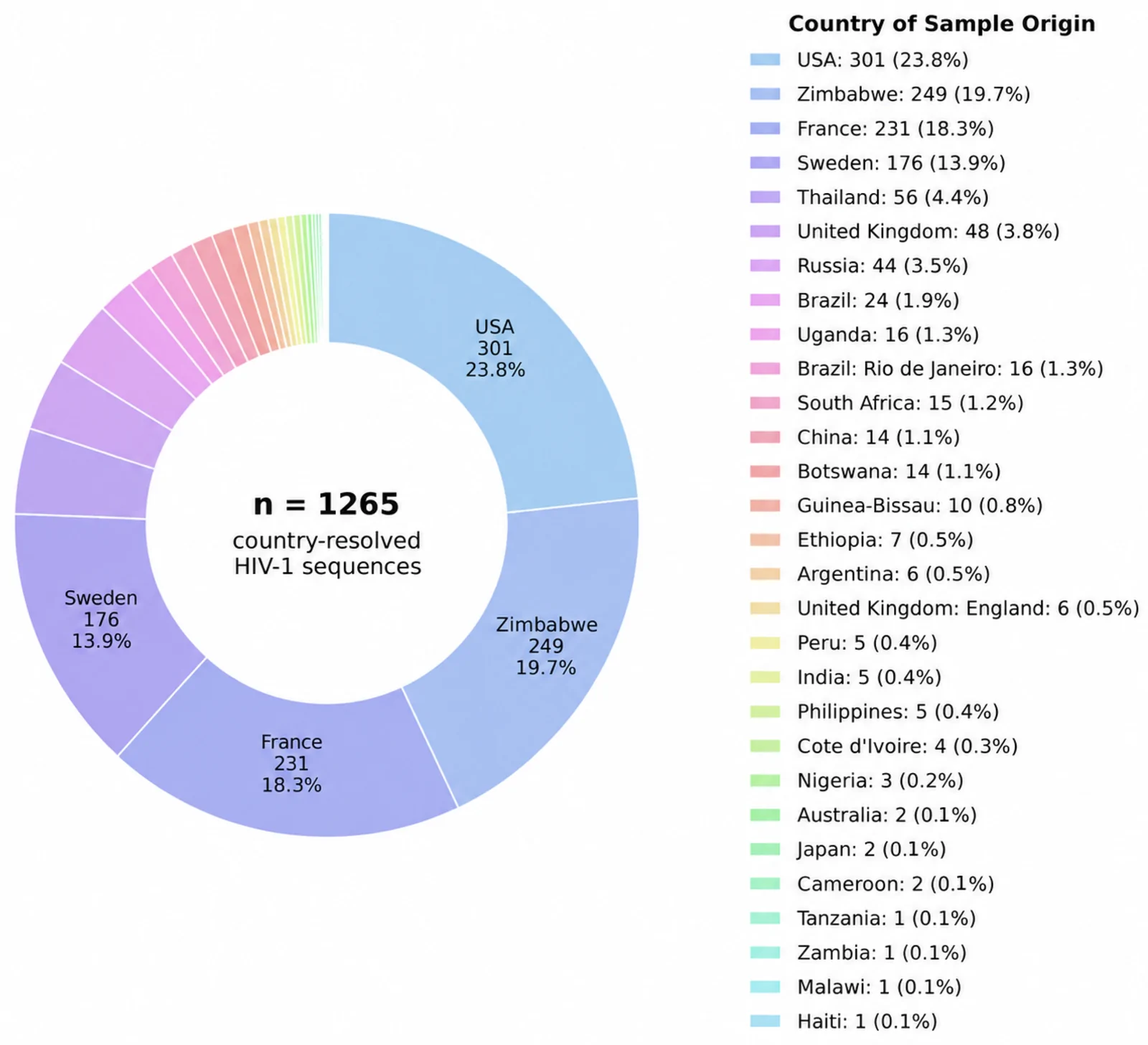
Distribution of HIV-1 sequences by country of sample origin. The donut chart shows the absolute number and proportion of records for which the country of sample origin was specified unambiguously. Aggregated geographic labels that did not correspond to a single country, including the “USA/Canada/Europe” category, as well as records without origin information, were not included in this chart. Therefore, the total number of records with a known country of sample origin was *n* = 1265.

The geographic origin of the samples was specified for 1804 records (99.7%), whereas it was missing for 5 records (0.3%). Percentages reported in this paragraph are calculated relative to the entire dataset (*n* = 1809). In total, the table contained 30 geographic labels, including individual countries as well as aggregated or more specific designations. The largest category was USA/Canada/Europe, with 539 records (29.8%). Unfortunately, the authors of these sequences did not specify the origin of individual samples and reported only the group of countries represented in their cohort. The next largest categories were USA, with 301 records (16.6%); Zimbabwe, with 249 records (13.8%); France, with 231 records (12.8%); and Sweden, with 176 records (9.7%; Figure 4).

**Figure 5:**
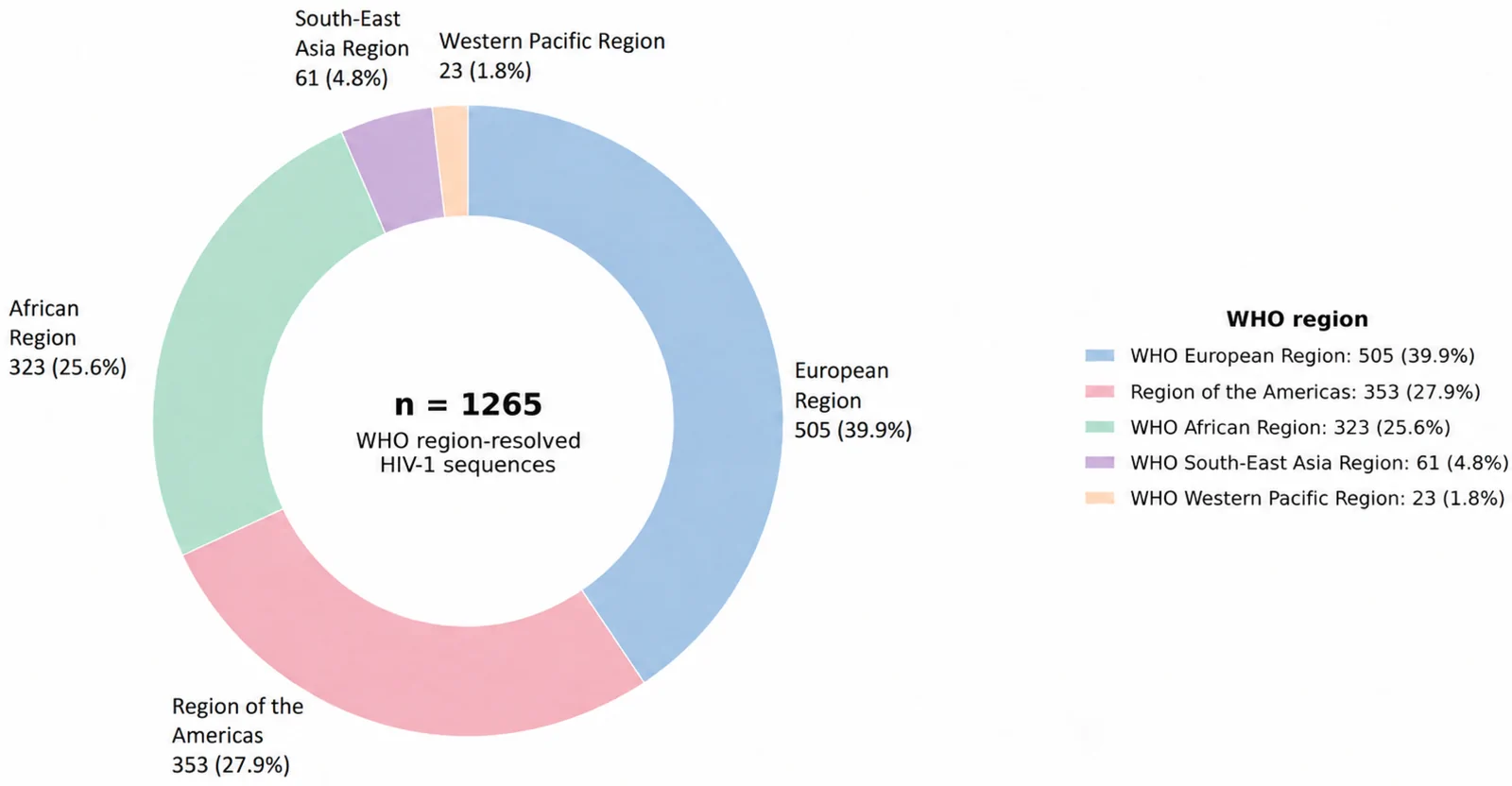
Distribution of HIV-1 isolate sequences with phenotypically determined tropism by WHO region. The donut chart shows the absolute number and proportion of records for which the geographic origin of the sample could be unambiguously assigned to one of the WHO regions.

We also assessed the geographic distribution of the dataset samples according to WHO regions. The aggregated USA/Canada/Europe category (*n* = 539) was not assigned to any individual WHO region because the source did not allow the country of origin of each sequence to be determined unambiguously. Among records that could be assigned unambiguously to a WHO region (*n* = 1265), the largest number belonged to the European Region, with 505 records (39.9%). This was followed by the Region of the Americas, with 353 records (27.9%); the African Region, with 323 records (25.6%); the South-East Asia Region, with 61 records (4.8%); and the Western Pacific Region, with 23 records (1.8%; Figure 5). No records in the final dataset could be unambiguously assigned to the Eastern Mediterranean Region.

### 2.5. Distribution of Samples by Completeness of HIV-1 Genome Sequencing

**Figure 6:**
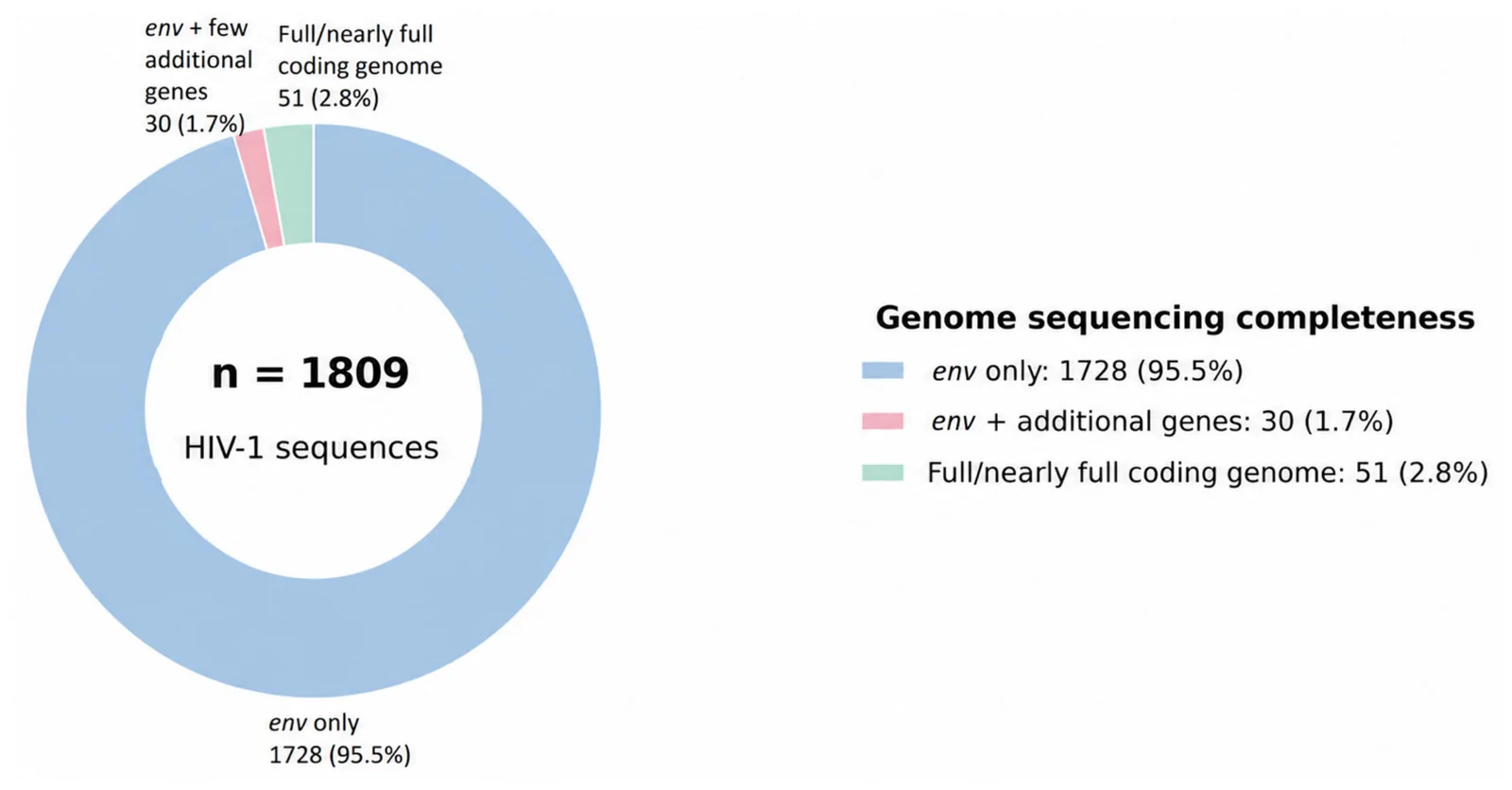
Distribution of HIV-1 sequences by genome sequencing completeness. The donut chart shows the proportion of records represented by the *env* gene only, records including *env* together with several additional HIV-1 coding genes, and complete or near-complete genome sequences containing all viral coding genes. For each category, the absolute number of records and the proportion of the total dataset are shown.

All collected sequences included the sequenced V3 region of gp120 in the HIV-1 *env* gene, because this region makes the greatest contribution to the determination of HIV tropism. Annotation limited to coding regions of this gene, without additional genomic regions, was present in 1728 records (95.5%; Figure 6). A small proportion of records represented longer genomic fragments that included the *env* gene together with other HIV-1 genes, including *gag*, *pol*, *vif*, *vpr*, *tat*, *rev*, *vpu*, and *nef* (Figure 6). Sequence lengths ranged from 81 to 9818 nucleotides.

## 3. Methods

### 3.1. Study Design

The present study was designed as a manual annotation and data integration project aimed at expanding the available set of HIV-1 sequences linked to experimentally determined tropism assessed by phenotypic methods. The resulting dataset was intended not as an alternative to the LANL database, but as a complement to it. The primary objective was to identify HIV-1 nucleotide sequences deposited in GenBank and described in the scientific literature for which tropism information was available, but which were absent from LANL at the time of writing.

Each record in the dataset corresponds to an HIV-1 sequence associated with a GenBank accession number, for which tropism information could be traced to a published phenotypic assay or to an author-provided GenBank annotation, provided that the study was conducted within a program that included phenotypic determination of tropism. The final resource was structured to support subsequent studies of HIV-1 coreceptor use, including the development and external validation of genotypic tropism prediction tools, machine learning-based sequence classification, analyses of the relationship between tropism and disease progression, and studies linking isolate tropism to the HIV-1 proviral landscape.

### 3.2. Literature Search Strategy

**Figure 7:**
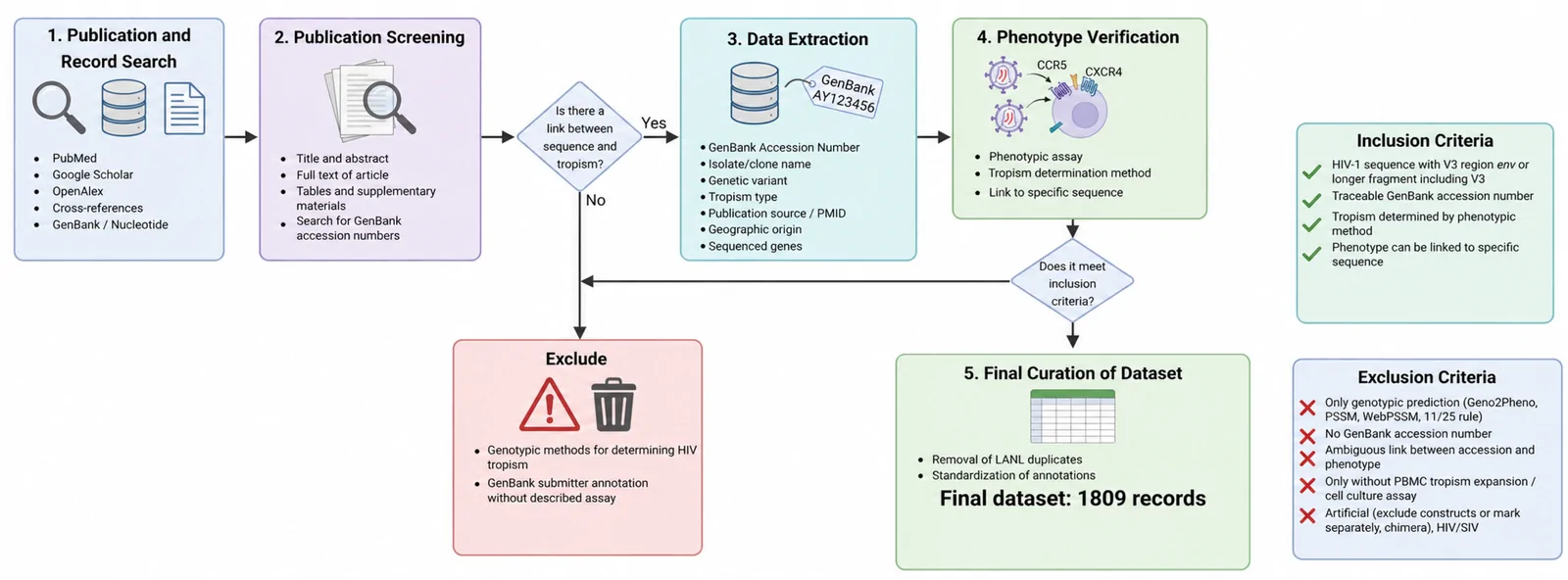
Pipeline for the search, verification, and selection of HIV-1 sequences for inclusion in the described dataset. The diagram shows the main steps of manual data curation: searching for publications and associated GenBank records, reviewing articles, tables, and Supplementary Materials, verifying the link between each specific sequence and tropism type, extracting metadata, checking the phenotyping method, and performing final record standardization. Only HIV-1 sequences with a traceable GenBank accession number were included in the dataset. These sequences had to contain the V3 region of the *env* gene, or a longer fragment including V3, and have phenotypically determined or explicitly annotated tropism that could be linked to the specific sequence. Records without a GenBank accession number, records with an ambiguous link between sequence and phenotype, records for which tropism was indicated only by genotypic prediction, and records without a separate coreceptor-use assay were excluded or flagged separately. After removal of LANL duplicates and standardization of annotations, the final dataset included 1809 records.

Publications were searched manually using the PubMed, Google Scholar, and OpenAlex search tools, as well as by cross-checking references in the identified articles and their associated GenBank records (Figure 7).

The main aim of the search was to identify studies in which HIV-1 sequences were linked to experimentally determined or explicitly annotated tropism. Search queries included combinations of terms related to HIV-1, the *env*/gp120/V3 genomic regions, coreceptor use, and phenotyping methods, including “HIV-1 tropism”, “HIV-1 coreceptor usage”, “CCR5”, “CXCR4”, “R5”, “X4”, “dual tropic”, “mixed tropism”, “V3 loop”, “gp120”, “*env*”, “phenotypic tropism assay”, “recombinant virus assay”, “pseudovirus assay”, “Trofile”, “PhenoSense”, “GHOST”, “U87”, “NP2”, “maraviroc resistance”, and “macrophage tropism”.

For each potentially eligible publication, the full-text article, tables, Supplementary Materials, and GenBank accession numbers reported in the study were examined. GenBank records associated with the identified publications were also checked. Earlier articles were additionally reviewed when the isolates, clones, or recombinant viruses under study had first been described in previous work. A publication was considered a potential data source if it allowed a specific sequence, or a group of sequences, to be linked to viral tropism, the method used to determine it, and, where possible, the source biological material or clinical context.

### 3.3. Inclusion Criteria

Records were included in the dataset if they met several mandatory criteria: (I) the sequence had to belong to HIV-1, have a traceable GenBank accession number, and contain the V3 region of the *env* gene or a longer genomic fragment including this region; (II) tropism information had to be available for the GenBank record, reported as R5, X4, R5/X4, dual/mixed tropism, CCR5 or CXCR4 use, or as a related phenotype provided that it had been experimentally determined and could be linked to the specific sequence; and (III) tropism information had to be traceable to a specific GenBank accession number through a published article, Supplementary Materials, the GenBank record, or a clearly described experimental context. Priority was given to sequences with phenotypically determined tropism obtained in cell-based coreceptor-use assays, recombinant virus systems, commercial Trofile/PhenoSense assays, or experiments using inhibitors of HIV binding to the CCR5/CXCR4 coreceptors.

### 3.4. Exclusion Criteria

Records were excluded from the dataset when a specific sequence could not be reliably linked to tropism information. Such cases included publications without GenBank accession numbers, records with a missing or ambiguous link between the accession number and phenotype, and sequences for which tropism was reported only as the result of genotypic prediction without confirmation by phenotypic methods. Records were also not included when they reported only virus cultivation in PBMCs or other cells without a separate coreceptor-use assay, because replication in culture alone was not considered evidence of tropism.

Records representing artificial constructs or chimeric viruses, such as HIV/SIV, were excluded separately as unsuitable for the main phenotypically confirmed dataset. Macrophage tropism was not used as a surrogate for coreceptor usage; R5, X4, or R5/X4 status was assigned only when direct information on CCR5 and/or CXCR4 use was available. After primary curation, all sequences already present in LANL were removed from the final table.

### 3.5. Data Visualization

Data processing and graph generation were performed using Python v3.12 [22]. The pandas library v3.0.3 was used for reading, cleaning, filtering, and aggregating tables [23]; NumPy v2.5.1 was used for numerical operations [24]; and matplotlib v3.11.0 and seaborn v0.3.12 were used for visualization [25,26].

## 4. Discussion

The presented dataset should primarily be considered a complementary phenotypically annotated set of HIV-1 isolate sequences, rather than an alternative to existing specialized databases. The main aim of the study was to compile sequences with a traceable link between genotype, phenotypically determined tropism, and publication source, including records that were absent from the main public search of reference datasets. In the context of HIV-1 research, this is particularly important because the development and external validation of genotypic tropism prediction tools still depend on the availability of machine learning data with reliable phenotypic indexing [2,14,15,16].

The vast majority of records in our dataset are represented by sequences including the V3 region of gp120 in the HIV-1 *env* gene. This reflects the established view that this region is the main molecular determinant of HIV-1 tropism. Specifically, the V3 loop of gp120 is considered such a determinant [27]. As a rule, nucleotide or amino acid sequences of this region are used for tropism prediction in most genotypic approaches [13,14,20,21,28]. At the same time, published data indicate that other regions of gp120, including V1, V2, C3, and the CD4-binding site, also contribute to this phenotype [29,30,31]. Another HIV envelope protein, gp41, may also be involved [32]. In addition, the accuracy of existing algorithms differs across viral genetic variants, especially for some non-B subtypes and CRFs. From this perspective, even the relatively small proportion of longer fragments that include *env* together with other genes, as well as near-complete genome sequences, is of practical interest. Such records may be used to develop machine learning models that take a broader genomic context into account.

The predominance of subtype B sequences in the dataset reflects the existing imbalance in accumulated data [33]. Subtype B is the most extensively studied HIV-1 variant, partly because it circulates in high-income countries, where research capacity is greater [34]. The larger number of collected records with R5 tropism also represents a challenge for machine learning and is driven by biological features of HIV-1. During transmission and early stages of disease, isolates using the CCR5 coreceptor usually predominate, whereas X4-tropic isolates are more often found at later stages of infection [35]. At present, a large proportion of HIV-positive patients receive antiretroviral therapy (ART) and have undetectable HIV viral loads [36], which further reduces the likelihood of detecting X4-tropic isolates.

Nevertheless, our dataset partially addresses this problem because it includes non-B subtypes (25.2%) as well as sequences of X4 and R5/X4 isolates (15.5% combined). This is important for machine learning and comparative bioinformatic analyses for two reasons. First, previous studies have shown that existing genotypic tools differ in accuracy across subtypes and CRFs [13]. Second, phenotypically confirmed X4- and R5/X4-tropic isolates, especially when they belong to non-B HIV-1 genetic variants, remain relatively rare material for model training and external validation.

Another potential direction for reuse of the dataset is related to studies of proviral reservoirs. Previous work has shown that, under the selective pressure of CCR5 antagonists, previously archived minor X4-tropic quasispecies can become detectable again [8].

Several technical limitations of this study should also be noted. The dataset remains imbalanced with respect to tropism, genetic variants, and sequencing completeness: it is dominated by R5-tropic isolates, HIV-1 subtype B sequences, and nucleotide sequences limited to the *env* gene. Geographic coverage is also uneven, and some sample origins are represented by aggregated labels that cannot be unambiguously assigned to a specific country or region. In addition, the annotation was based on published data and GenBank records rather than on repeated experimental phenotyping of all sequences. It should be emphasized again that this work does not represent an independent database, but rather a small addition to the existing collection of phenotyped sequences compiled in LANL, increasing it by approximately 11% at the time of writing.

Nevertheless, the collected dataset may be useful for the development of new methods for genotypic tropism prediction and for studies related to the clinical course of HIV infection and the landscape of proviral reservoirs.

## 5. Conclusions

In the present study, a non-redundant dataset of HIV-1 nucleotide sequences linked to phenotypically determined tropism was created. The final dataset includes 1809 records, each associated with a GenBank accession number and accompanied by key metadata, including the viral genetic variant, tropism type, scientific source, assay type used, publication data, geographic origin of the sample, and the list of sequenced genes. Most records were represented by *env* gene sequences and R5-tropic isolates; however, the dataset also includes non-B HIV-1 genetic variants, recombinant forms, and X4 and R5/X4 variants, increasing its potential value for subsequent comparative and computational studies.

The created resource should not be considered an independent alternative to existing databases, but rather a small curated supplement intended for reuse in genotypic tropism prediction, machine learning, external validation of predictive models, and studies related to HIV disease progression and proviral reservoirs. The main limitations of the dataset are associated with imbalances in tropism, subtypes, geographic origin, sequencing completeness, and the heterogeneity of the original phenotypic assays. Nevertheless, the clear and traceable link between sequence, phenotype, and data source makes this dataset a useful complementary resource for the further development and validation of new bioinformatic approaches to studying HIV-1 tropism.

## Figures and Tables

**Table 1:** General characteristics of the generated dataset.

Dataset Characteristic	Value
Total records	1809
Records with PMID	1527
Records without PMID	282
Unique PMIDs	32
Records with a publication/source title	1531
Unique publication/source titles	33
Number of unique genetic variants (subtypes, CRFs, URFs)	20
Subtype B records	1354
Non-B subtype records	455
CRF/URF records	103
R5 records	1528
R5/X4 records	164
X4 records	117
Phenotypic assay categories	14
Records with specified geographic origin	1804
Records without specified geographic origin	5
Records containing only the *env* gene	1728

## Data Availability

The data presented in this study are openly available in Zenodo at https://zenodo.org/records/23163974 (accessed on 1 October 2026), reference number (DOI) 10.5281/zenodo.23163974. Additional information can be obtained from the corresponding author upon request.

## Abbreviations

The following abbreviations are used in this manuscript:

ART	Antiretroviral therapy
CCR5	C-C chemokine receptor type 5
CRF	Circulating recombinant form
CXCR4	C-X-C chemokine receptor type 4
DNA	Deoxyribonucleic acid
FDA	Food and Drug Administration
GHOST	Green fluorescent protein (GFP)-expressing Human Osteosarcoma cells
HIV-1	Human immunodeficiency virus type 1
LANL	Los Alamos National Laboratory
NP2	Human glioma cell line
PBMC	Peripheral blood mononuclear cell
PMID	PubMed Identifier
RNA	Ribonucleic acid
URF	Unique recombinant form
WHO	World Health Organization
